# HIV testing services in healthcare facilities in South Africa: a missed opportunity

**DOI:** 10.1002/jia2.25367

**Published:** 2019-10-10

**Authors:** Tonderai Mabuto, Bhakti Hansoti, Deanna Kerrigan, Nolundi Mshweshwe‐Pakela, Griffiths Kubeka, Salome Charalambous, Christopher Hoffmann

**Affiliations:** ^1^ Implementation Research Division The Aurum Institute Johannesburg South Africa; ^2^ The University of the Witwatersrand School of Public Health Johannesburg South Africa; ^3^ Department of Emergency Medicine Johns Hopkins School of Medicine Baltimore MD USA; ^4^ Department of International Health Johns Hopkins Bloomberg School of Public Health Baltimore MD USA; ^5^ Department of Sociology American University Washington DC USA; ^6^ Department of Medicine Johns Hopkins University School of Medicine Baltimore MD USA; ^7^ Department of Health, Behavior, and Society Johns Hopkins Bloomberg School of Public Health Baltimore MD USA

**Keywords:** HIV, South Africa, facility‐based testing, primary care, public health, testing, implementation science

## Abstract

**Introduction:**

South Africa (SA) has the world's highest burden of HIV infection (approximately 7.2 million), yet it is estimated that 23.5% women and 31.5% of men are unaware that they are living with HIV. The 2015 national South African HIV testing guidelines mandate the universal offer of HIV testing services (HTS) in all healthcare facilities.

**Methods:**

A multi‐prong approach was used from January 2017 to June 2017 to evaluate the current implementation of HTS in ten facilities in the Ekurhuleni District of SA. First, we conducted patient exit interviews to quantify engagement in HTS services. Second, we systematically mapped the flow of individual patients through the clinic.

**Results:**

We conducted a total of 2989 exit interviews and followed 568 patients for value stream mapping. Overall self‐reported testing acceptance was high at 84.7% (244), but <10% of the patients (288) were offered testing. Female patients were more likely to be offered testing (233/2046, 11.4% vs. 55/943, 5.8% in males; chi‐square *p* < 0.005), and also more likely to accept testing (203/233, 87.1% vs. 41/55, 74.6% in males; chi‐square *p* = 0.02). Value stream mapping revealed that patients offered HIV testing had a total visit time of 51 minutes more (95% CI: 30‐72) compared to those not offered testing.

**Conclusions:**

The poor delivery of HTS appears to be due to a failure to recommend HTS and the added time burden placed on those accepting testing. There were significant differences in both the offer and acceptance of testing by gender. Health system issues need to be addressed to improve HTS delivery.

## 1 Introduction

HIV infection contributes to half of the deaths in South Africa (SA), with the highest mortality among those who are severely immunocompromised and not yet receiving antiretroviral therapy (ART) 1, 2. Early identification of people living with HIV and subsequent engagement into HIV treatment is essential, to reducing illness, death and HIV transmission 3, 4, 5. Furthermore, early diagnosis is key to maximizing the potential impact of universal test and treat approaches. As an entry point into the HIV care continuum, the optimal delivery of HIV testing services (HTS) is particularly relevant for HIV programmes in SA which aim to initiate an additional 2.1 million people onto ART by the end of December 2020 6, 7, 8. Given the current national estimates of 20% to 30% of undiagnosed HIV infection; approximately 420,000 to 600,000 new HIV diagnoses are required to meet these ART initiation targets 9.

HIV programmes in SA employ a strategic mix of modalities to promote universal and equitable access to HTS. Of these approaches, health facility‐based HTS is a longstanding and essential method for identifying individuals living with HIV and for providing annual repeat testing for those who are negative and linkage to HIV prevention services. Compared to community‐based HTS, facility‐based testing, due to higher patient volumes and the convenience of a single location, is more likely to reach higher testing numbers and has the ability to provide onsite ART initiation and follow‐up services. In the recent years, facility‐based testing has shifted towards more proactive approaches in which clinic staff offer HTS to clinic attendees who may not be seeking HIV care services – this is commonly referred to as “provider‐initiated counselling and testing” (PICT). Several studies have shown high acceptability, high uptake and a high yield of newly identified HIV infected individuals, through this approach 10, 11, 12, 13. More importantly, PICT reaches individuals who are already seeking clinical care and possibly, for this reason, individuals diagnosed through facility‐based HTS are more likely to continue into HIV care compared to those diagnosed through community‐based HTS 14, 15, 16. Unfortunately, PICT remains highly underutilized in many South African health facilities, despite a national policy mandating its offer as a standard component of medical care 17. Several barriers which limit the translation of PICT policies into routine practice have been documented: the lack of human resources, inadequate infrastructure to ensure privacy and norms among clinic staff which inhibit the embrace of PICT into routine clinical practice. Understanding the dynamics of HTS across different types of health facilities and the critical constraints to its optimal delivery can allow for increased use of HTS as a crucial public health tool.

In this study, we sought to describe the current delivery of HTS services within a diverse group of health facilities in Ekurhuleni District, the fourth largest metropolitan city in SA. We also sought to characterize missed opportunities and current constraints on delivering HTS by systematically identifying the most probable or highest impact failures within a health facility system to optimally deliver HTS.

## 2 Methods

### 2.1 Study design

**Figure 1:**
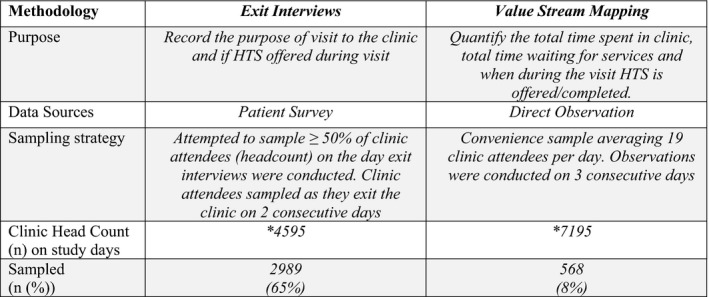
Facility‐based HIV testing evaluation constructs *Represents total clinic head counts for all clinics on the day the exit interviews or value stream mapping activities were conducted. The two activities were conducted on separate days. HTS, HIV testing services.

This was a cross‐sectional study, conducted from January 2017 to June 2017, in ten public sector health facilities in the Ekurhuleni District of SA. This district has the second‐highest district‐level HIV prevalence (14.3%) in SA and is comprised of urban and peri‐urban settings 18, 19. The study sites consisted of six primary health care centers (PHC), three community health care centers (CHC) and one district‐level hospital. The PHCs are open Monday to Friday for eight hours a day and run by nurses and clerks, who manage care with limited to no physician oversight. CHCs are open 24 hours a day and have onsite physician support. There are 93 public health clinics and six public hospitals in Erkhuleni District. Facilities were selected in coordination with the district‐level Department of Health to achieve geographic diversity including distribution across North, South and Eastern sub‐districts. All facilities included in the study had HIV counselling and testing personnel (lay counsellors) and routinely provide HTS free of charge according to the 2015 National HIV counselling and testing (HCT) guidelines 17. Under these guidelines, HIV testing is based on an opt‐in approach requiring informed consent with pre‐ and post‐test counselling in a confidential private setting. HIV testing follows an algorithm based on approved rapid point‐of‐care blood tests that use finger‐prick capillary blood samples. Specifically, we sought to describe: (1) the delivery of facility‐based HTS using exit interviews; and (2) the impact of facility‐based HTS on clinic wait time using value stream mapping (Figure 1).

### 2.2 Outcome measures

We sought to describe (1) the proportion of clinic patients offered testing; (2) the proportion offered testing who underwent HTS; and (3) service and wait times during a clinic visit.

### 2.3 Data collection

We used two streams of data collection that were conducted in parallel within same facilities. However, each of the data collection streams were independent from one another and occurred on different days.

Data from the exit interviews were conducted using a survey administered by an interviewer to patients as they exited the clinic over the course of the day. An effort was made to approach and interview all adult patients (who appeared to be aged ≥ 18 years) at the end of their clinic visit as they exited the clinic. However, due to high patient volume and the size of the study team we sought to sample at least 50% of the total clinic daily headcount. The total headcount was collected from the daily clinic records. Notably, we did invite all individuals exiting the clinic to participate, including individuals who did and did not complete the primary purpose of the clinic visit. This allowed us to include anyone coming to a clinic for services and not offered HTS as a missed HTS opportunity, whether or not they received other intended services. All data were collected by four trained study staff who were experienced in recruiting research participants and collecting research data. Exit interviews were conducted on two consecutive days at each facility to include day to day variability in‐clinic use. Prior to survey administration, study staff asked clinic attendees for their age; persons < 18 years were ineligible to participate due to additional requirements of parental/guardian consent. No additional information was documented for patients who declined participation in the exit interviews. In addition to basic demographic data such as age and sex, patients were asked their reason for visit, if they were offered an HIV test, when during the clinic visit the HIV test was offered and conducted, their reasons for declining HIV testing and if they had received an HIV test in the 12 months prior to their clinic visit. Participants provided oral consent for the exit interviews.

The value stream mapping data were collected by tracking and manually time‐stamping the entire clinic pathway for each participant from start to finish. A convenience sample of patients were approached over the sampling days and offered participation. Study staff arrived at the health facility before clinical services commenced, mostly before 8:00 am, and gave a group talk about the value stream mapping activity which covered the purpose of the study, procedures, eligibility criteria (clinic attendees aged ≥ 18 years) and the process of selection with the aim of balancing the sample on sex. Following the study brief, two of the four study staff randomly approached three to four females each, while the other two study staff approached a similar number of males. In addition, study staff divided themselves to select patients from both the front and back ends of the queue. At the time of approaching patients in the queue, study staff did not ask patients to provide their reason for visiting the health facility. This information was only collected after patients had provided written consent to participate.

The study staff assumed a “peripheral observer role,” they did not interfere with clinic procedures or observe clinical interactions. In addition, clinic staffs were unaware of which patients had been enrolled for value stream mapping. The timesheet was completed for each participant and included details such as arrival times, wait times, accessed service points, service times, HIV testing service and departure times. Participants provided oral consent for participation in the value stream mapping.

### 2.4 Statistical analyses

We calculated a sample size for the exit interviews to provide an estimate of coverage of offering HTS with an accuracy of 15% assuming offering to 20% of clients and an alpha of 0.05 and 80% power. A convenience sample of 30 to 50 patients per facility was selected for value stream mapping, without *a priori* sample size calculations. Service time was calculated as all time spent interacting with clinic staff, including registering at reception, time retrieving files, vital signs, clinician consultation, phlebotomy and check‐out procedures. Wait time was recorded as the time that the participant was in a queue after completing one step of the clinic visit and prior to the next step. In descriptive analyses, categorical variables and measures of HTS coverage and uptake were summarized using frequencies (n) and percentages (%). Continuous variables, including service and wait times, were summarized using median and interquartile ranges (IQR). In secondary analyses, two‐way comparisons for the frequency and distribution of patient engagement in HTS by sex were conducted using the Pearson's chi‐square test. Results were provided using summary statistics and chi‐square testing to assess for correlations between sex data. Data were analysed using STATA v.12 (StataCorp, LLC, College Station, TX, USA).

### 2.5 Ethical considerations

The study was approved by the University of the Witwatersrand Human Research Ethics Committee, the Johns Hopkins University School of Medicine Institutional Review Board, and the Ekurhuleni District Research Committee.

## 3 Results

During the sampling time frame, 2989 patients completed exit interviews, and 568 patients were observed to complete value stream mapping. Based on the demographics of those interviewed the median age was 36 years (IQR 29‐46 years) and the majority of patients were female (2046/2989, 68.5%). The plurality of females were aged 30‐40 years (34.5%, 706/2046). The plurality of men were aged 40‐50 years (33.5%, 316/943).

### 3.1 Reason for clinic visits

On the days that exit interviews were conducted, 4595 patients attended the healthcare facilities, of which 2989 (65.9%) participated in exit interviews. The majority of patients (780, 29.8%) presented for health maintenance and chronic health services. A smaller percentage, 449 (17.2%) of patients presented for care for acute services, generally representing care for an acute injury or illness. A small percentage of visits were for paediatric patients accompanied by an adult guardian (only the guardian was interviewed) with 415 (15.9%) of patients presenting for child health services (Table 1). Most of the patients reported that they had prior visits to the same clinic (2804, 93.8%). Table 1 presents the reason for visit, the percentage offered testing and the percentage who accepted testing by visit.

**Table 1:** Reason for visit by clinic type (N = 2989)

Reason	Offered testing n (%)/total	Accepted testing n (%)/offered	Total N
Chronic care	8 (0.9)	4 (80.0)	910
Acute care	68 (13.4)	44 (64.7)	506
Female health	165 (36.2)	157 (95.2)	456
Male health	5 (16.7)	4 (80.0)	30
Child health	7 (1.6)	4 (57.1)	437
TB services	14 (8.8)	13 (92.9)	159
Pharmacy	1 (0.3)	1 (100)	305
Investigations	5 (16.1)	5 (100)	31
Other	15 (9.7)	13 (86.8)	155
Total (n)	288 (9.9)	244 (84.7)	

### 3.2 HTS offer and acceptance

Of those interviewed, 1548 (51.8%) patients reported that they had not received an HIV test in the last 12 months. However, only 288 (9.6%; IQR 8.0‐12.2%) clients were offered HIV testing. Only 64 (4.4%; IQR 1.8‐8.3%) of patients not tested in the last 12 months were offered testing. Accepting testing when offered was 84.4% (244/288). Both the proportion offered and proportion accepting testing varied by reason for clinic visit (Table 1). The offer for testing was highest in female health services (which included family planning and care for sexually transmitted infections) with 36.2% (165/456) offered HTS. Testing acceptance was highest in female health (95.2%) and TB services (92.9%). Among patients presenting with an acute care complaint 69.6% (16/23) patients accepted HTS when offered. Among men coming for voluntary male medical circumcision 16.7% (5/30) were offered an HIV test, of which 80% (4) accepted. A total of 44 patients refused testing; the main reasons were already knowing their status (25/44, 55.6%), being in a hurry (8/44, 17.8%), and not being ready to know their status (7/44, 15.6%). When comparing the offer and acceptance of testing by sex, we found that female patients were more likely to be offered testing (233/2046, 11.4% vs. 55/943, 5.8% in males; chi‐square *p* < 0.005). Testing acceptance was also higher for women (203/233, 87.1% vs. 41/55, 74.6% in males; chi‐square *p* = 0.02) (Table 2).

**Table 2:** Patient report of HIV testing services in primary healthcare facility (N = 2989)

	Males n (%)	Females n (%)	*p*‐Value	Total N
Asked about HIV testing	107 (11.4)	387 (18.9)	<0.005	494
Reported being tested in the last 12 months	359 (38.1)	1082 (52.9)	<0.005	1441
Offered testing	55 (5.8)	233 (11.4)	<0.005	288
Accepted testing if offered	41 (74.6)	203 (87.1)	0.020	244
Total n (%)	943 (32.5)	2046 (67.5)		2989

### 3.3 Service and wait times by HTS engagement

Across all sites a total of 568 patients were followed from the start to the end of their clinic visit. Among these patients, 68 patients (12.0%) were offered HTS, of which 42 accepted (61.7%) testing. The median visit time was 128 minutes (IQR: 87,202), with only 20 minutes spent receiving services (IQR: 10, 36) and 78 minutes waiting (IQR: 39,137) (Table 3). Patients who accepted HTS had longer total visit times (200 minutes; IQR: 145,270) compared to those who did not undergo HTS (119 minutes; IQR 81,197) (Table 3).

**Table 3:** Visit time, service time and wait time presented as a median with IQR by offer or completion of HTS services (N = 568)

	HTS offered	HTS not offered	HTS done	HTS not done	All
Total visit time (median minutes [IQR])	160 [135,249]	119 [81,197]	200 [145,270]	122 [83,197]	128 [87,202]
Total service time (median minutes [IQR])	51 [29, 70]	17 [9, 31]	62 [32, 75]	18 [10, 32]	20 [10, 36]
Total wait time (median minutes [IQR])	52 [52,153]	76 [37,133]	106 [43,155]	77 [38,133]	78 [39,137]
Proportion of value‐added time (%)	32	14	31	15	16
Total (n)	68	500	42	526	568

IQR, interquartile range; HTS, HIV testing services.

**Figure 2:**
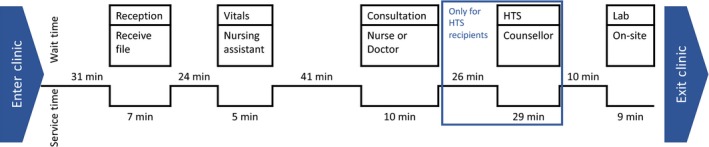
Schematic overview of median wait time, per service, during visits using value stream mapping HTS, HIV testing services.

In part, the increase in total visit time for patients who accepted HCT was due to the time it took to undertake HIV testing. Patients spent a median of 29 minutes (IQR: 18, 43) in direct contact with a HTS counselling when undergoing HTS, compared to under 10 minutes (IQR: 5, 17) direct contact with a clinic member (e.g. clerk, clinician) for all other services. Figure 2 presents a schematic overview of how an individual patient's time was spent during the clinic visit. Throughout their visit, patients spent a significant amount of time waiting for services with at least four wait periods, each often longer than 10 minutes. Patients who did undergo HTS almost always did this as an add‐on to the visit, after completing all other visit components.

Most patients were offered HTS by the HTS counsellor (34, 50%) or during provider consultation (23, 34%), the remainder were unknown. Only three patients were offered HTS in the waiting areas, potentially allowing them to receive a value‐added service during the waiting period. The majority of patients received HTS from the HTS counsellor (32, 84%).

## 4 Discussion

This study presents a detailed description of HTS delivery in 10 healthcare facilities in SA. We observed that <10% of clinic patients were offered HTS. However, in those who HTS was offered, a large proportion reported accepting testing (over 80%). The value stream mapping identified that the timing of HTS service delivery did not take advantage of considerable queueing time in the clinic. HTS was generally offered later during a patient's long visit time and added further to the number of hours a patient spent at the clinic. This represents missed opportunities for offering HTS and for integrating HTS into the value stream of a normal clinic visit.

The majority (70%) of South Africans who receive HTS reported accessing HTS through public‐sector primary health clinics. (19) This highlights the importance of clinics in reaching individuals with HTS and increasing the proportion of people with HIV who know their status. In a recent South African study across 67 health facilities, 18% of clinic attendees had never tested for HIV. (20) Unless changes are made to HTS delivery in facilities like those we observed, these individuals may remain untested. Overall acceptance of HTS, when offered, appeared comparable to 73% uptake (95% CI: 55‐87) of facility‐based HIV testing, pooled across 12 health facilities in sub‐Saharan Africa 16.

In addition to HTS uptake, the yield of HIV positive individuals identified through HTS is an indicator of the efficiency when the goal is to diagnose HIV and rapidly initiate ART. In this regard, facility‐based HTS approaches are generally more efficient in identifying a higher yield of HIV positive individuals (18‐20%) compared to community‐based approaches for the general population (6‐11%) 16.

The translation of policy guidance on facility‐based testing into practice is fraught with several operational challenges. Importantly, HTS delivery in overstretched public‐sector healthcare facilities is time‐consuming, as currently delivered, and requires substantial human resources. Even the dedicated cadre of HTS personnel (lay counsellors) were only able to provide testing to a limited number of patients per day. A recent evaluation of the HIV lay counselling and testing profession in SA interviewed 32 lay counsellors from 62 health facilities. Staff self‐reported counselling an average of 12 clients per day and testing only 9‐25 clients on busy clinics days, which is a fraction of the total number of patients that present for care 20, 22. Due to the time demands of providing HTS, clinicians provided only a small proportion of HTS sessions (15%), similar to prior observations 21.

Our study also found that there was a cost at the patient level for undertaking HTS. Engaging in HTS considerably increased the visit time for the patients in our study. Queueing, waiting for a rapid test to result, and undergoing pre‐test and post‐test counselling all contribute to the time. In our study, patients engaged in HTS had an additional waiting time of an hour compared to those who did not engage in HTS. Long clinic wait times without receiving services have been shown to lead to poor engagement in HIV care services as a result of the high opportunity costs associated with accessing these services 26. However, when offered early during the clinic visit, the additional time costs of HTS engagement may be mitigated. Findings from a study of 36 health facilities in SA, Tanzania and Uganda, showed that HTS delivery by counsellors or clinicians before clinical consultation had the highest uptake compared to HTS during or after clinical consultation 23.

We found the use of a systems engineering study design useful in identifying barriers to HTS delivery beyond patient‐level factors for not accepting testing 24, 25, 26, 27. Value stream mapping identified inefficiencies (“waste”) within the clinic pathway that can be targeted to improve HTS uptake. Additional interventions for improving the efficiency of HTS delivery within the constraints of limited human resources and adequate workspace, include abbreviated pre‐ and post‐test counselling, and the integration of HIV self‐testing options into facility‐based HTS approaches to triage out HIV negative patients. Once system efficiencies have been introduced into clinic pathways, health promotion activities need to be intensified to increase awareness and uptake of HTS.

### 4.1 Limitations

This study has the strength of assessing multiple clinics providing routine service delivery in a real‐world setting without augmentation from research or academic staff. There are some limitations. First our study was conducted in a single semi‐urban/metropolitan district in SA, and thus the generalizability of the results may be limited, especially to more rural districts or low volume clinics. Second, our study utilized a convenience sample, which captured close to two‐thirds of clinic visit attendees on the days of data collection. This may result in sampling bias, however due to the limitations of the clinic system it was not possible to gather information on those that were missed by our sampling strategy. Third, our study sampled the clinics on randomly chosen days over a six‐month period, which may introduce a bias due to variances in staffing; however, an analysis of variance revealed no significant differences in offer of HIV testing by clinic type. In addition, the majority of the data were collected prior to winter months when there is typically an increase in health care use. Lastly, the use of self‐report may have led to reporting bias, potentially leading to an underestimation of the proportion offered testing (if the offer of testing is perceived to be associated with bad behaviour) and over‐estimating the proportion that accepted; furthermore the estimated proportion offered testing does not account for the fact that a proportion of people accessing services already know that they are HIV positive.

## 5 Conclusions

The current provision of HIV services in healthcare facilities reached a small proportion of potentially eligible clinic patients. This is a population that is already seeking care allowing for both efficient provision of HTS and is associated with higher rates of linkage to care following testing positive. Thus, the current state of facility‐based HTS falls short of the potential. Identifying approaches to increase facility‐based HTS may provide a feasible and sustainable approach to improved HIV testing for part of the population of people with HIV who are currently not in care or receiving ART.

## Competing interests

The authors declare that they have no conflict of interest.

## Authors’ contributions

TM, CH, DK and SC, designed the research study. TM, NMP, GK and SC, performed the research. BH and CH, analysed the data. BH, TM and CH, wrote the paper. All authors have read and approved the final manuscript.

### Funding

The study was funded by the United States Agency for International Development (USAID).

## Supporting information


**Table S1.** Summary statistics of exit interviews by study site (N = 2989).
