# Supplementary material for: HIV testing services in healthcare facilities in South Africa: a missed opportunity
Source: J Int AIDS Soc. 2019 Oct 10;22(10):e25367. doi: 10.1002/jia2.25367 (PMC6785782; doi:10.1002/jia2.25367)
Supplement: Supplementary file 1 — Table S1. Summary statistics of exit interviews by study site (N = 2989). [file JIA2-22-e25367-s001.docx]

Supplemental Table 1: Summary statistics of exit interviews by study site (N=2989)

|  | Bertha Gxowa | Ethafeni | Itireleng | Katelehong | Mary Moodley | Nokuthela Ngwenya | Ramon  -okopi | Selope Thema | Sonto Thobela | Wannen  -berg | **Total** |
| --- | --- | --- | --- | --- | --- | --- | --- | --- | --- | --- | --- |
| Facility Type | Hospital | PHC | PHC | PHC | CHC | CHC | CHC | PHC | PHC | PHC |  |
| Clinic Headcount (N) | 394 | 436 | 614 | 444 | 481 | 599 | 549 | 351 | 290 | 437 | **4595** |
| Total Captured  n (%) | 373  (94.7%) | 238  (54.6%) | 350  (57.0%) | 312  (70.3%) | 253  (52.6%) | 349  (58.3%) | 365  (66.5%) | 257  (73.2%) | 186  (64.1%) | 306  (70.0%) | **2989**  **(65.0%)** |
| Mean age (years)* | 40.4 | 38.6 | 37.8 | 38.9 | 38.8 | 39.7 | 37.1 | 38.7 | 39.1 | 36.3 | **38.5** |
| Male  n (%) | 144  (38.6%) | 52  (21.9%) | 122  (34.9%) | 88  (28.2%) | 108  (42.7%) | 116  (33.2%) | 104  (28.5%) | 62  (24.1%) | 58  (31.2%) | 89  (29.1%) | **943**  **(31.6%)** |
| First time at clinic  n (%) | 42  (11.3%) | 7  (2.9%) | 19  (5.4%) | 27  (8.6%) | 14  (5.5%) | 19  (5.4%) | 28  (7.7%) | 11  (4.3%) | 8  (4.3%) | 10  (3.3%) | **185**  **(6.2%)** |
| Unscheduled visit  n (%) | 104  (27.9%) | 108  (40.9%) | 124  (40.5%) | 85  (27.4%) | 52  (20.6%) | 89  (25.5%) | 111  (30.4%) | 59  (22.9%) | 41  (22.0%) | 77  (25.2%) | **737**  **(24.7%)** |
| Asked about HIV test  n (%) | 51  (13.7%) | 25  (10.5%) | 51  (14.6%) | 75  (24.0%) | 9  (3.6%) | 94  (26.9%) | 53  (14.5%) | 33  (12.8%) | 36  (19.4%) | 67  (21.9%) | **494**  **(16.5%)** |
| Tested in last 12 months n (%) | 152  (40.8%) | 108  (45.4%) | 158  (45.1%) | 172  (55.1%) | 96  (37.9%) | 181  (51.9%) | 199  (54.5%) | 113  (43.9%) | 88  (47.3%) | 174  (56.9%) | **1441**  **(48.2%)** |
| Offered testing  n (%) | 31  (8.3%) | 19  (8.0%) | 39  (11.1%) | 38  (12.2%) | 9  (3.6%) | 61  (17.5%) | 20  (5.5%) | 25  (9.7%) | 23  (12.4%) | 23  (7.5%) | **288**  **(9.6%)** |
| Eligible offered test*  n (%) | 7  (3.2%) | 1  (0.8%) | 6  (3.1%) | 15  (10.7%) | 0  (0%) | 14  (8.3%) | 3  (1.8%) | 4  (2.8%) | 2  (2.0%) | 12  (9.1%) | **64**  **(4.1%)** |
| Accept if offered**  n (%) | 28  (90.3%) | 16  (84.2%) | 34  (87.2%) | 29  (76.3%) | 9  (100.0%) | 49  (80.3%) | 18  (80.3%) | 22  (88.0%) | 21  (91.3%) | 18  (78.3%) | **244**  **(84.7%)** |

* Eligible = Those patients that reported that they had not been tested for HIV in the last 12 months

** Of those that are offered a HIV test percentage that accept
